# Brain Glucose Metabolism in Health, Obesity, and Cognitive Decline—Does Insulin Have Anything to Do with It? A Narrative Review

**DOI:** 10.3390/jcm10071532

**Published:** 2021-04-06

**Authors:** Eleni Rebelos, Juha O. Rinne, Pirjo Nuutila, Laura L. Ekblad

**Affiliations:** 1 Turku PET Centre, University of Turku and Turku University Hospital, 20520 Turku, Finland; eleni.rebelos@utu.fi (E.R.); jurin@utu.fi (J.O.R.); pirjo.nuutila@utu.fi (P.N.); 2 Department of Endocrinology, Turku University Hospital, 20520 Turku, Finland

**Keywords:** brain glucose uptake, positron emission tomography, insulin sensitivity, Alzheimer’s disease

## Abstract

Imaging brain glucose metabolism with fluorine-labelled fluorodeoxyglucose ([^18^F]-FDG) positron emission tomography (PET) has long been utilized to aid the diagnosis of memory disorders, in particular in differentiating Alzheimer’s disease (AD) from other neurological conditions causing cognitive decline. The interest for studying brain glucose metabolism in the context of metabolic disorders has arisen more recently. Obesity and type 2 diabetes—two diseases characterized by systemic insulin resistance—are associated with an increased risk for AD. Along with the well-defined patterns of fasting [^18^F]-FDG-PET changes that occur in AD, recent evidence has shown alterations in fasting and insulin-stimulated brain glucose metabolism also in obesity and systemic insulin resistance. Thus, it is important to clarify whether changes in brain glucose metabolism are just an epiphenomenon of the pathophysiology of the metabolic and neurologic disorders, or a crucial determinant of their pathophysiologic cascade. In this review, we discuss the current knowledge regarding alterations in brain glucose metabolism, studied with [^18^F]-FDG-PET from metabolic disorders to AD, with a special focus on how manipulation of insulin levels affects brain glucose metabolism in health and in systemic insulin resistance. A better understanding of alterations in brain glucose metabolism in health, obesity, and neurodegeneration, and the relationships between insulin resistance and central nervous system glucose metabolism may be an important step for the battle against metabolic and cognitive disorders.

## 1. Introduction

The incidence and prevalence of obesity and type 2 diabetes (T2D) have reached epidemic dimensions [1,2]. Both obesity and T2D have been linked to an increased risk of several neurodegenerative disorders, including the most prevalent form of dementia, Alzheimer’s disease (AD) [3,4], but the exact pathophysiological mechanisms that link obesity and T2D to AD are not yet clear. Obesity is closely associated with insulin resistance (IR), the pathophysiological hallmark of the metabolic syndrome and T2D [5]. In recent years the demonstration of IR in the brains of AD patients [6] and the association between IR and cognitive decline [7,8], and dementia [9,10,11] has led to the hypothesis that IR could represent a link between T2D and AD. However, many open questions remain, in particular considering the possible relationship between systemic IR and brain glucose uptake (BGU), measured with fluorine-labeled fluorodeoxyglucose positron emission tomography [^18^F]-FDG-PET, and how these alterations in BGU might affect or reflect neurodegeneration and the neuropathological changes of AD. At present, the association between IR and cerebral changes is being studied both by groups focusing on metabolic disorders in middle-aged or young subjects [12,13,14,15] and by groups specialized in dementia research [6,16,17,18,19,20]. In this review, we aim to combine results from both lines of research in an attempt to clarify the latest results evaluating the association between peripheral IR and brain glucose uptake, with a special emphasis on the interpretation of the differences in findings from [^18^F]-FDG-PET scans performed in the fasting state and during an insulin-stimulated state, i.e., the hyperinsulinemic euglycemic clamp. Even though our review is mainly focused on [^18^F]-FDG-PET findings, we acknowledge that several other neuroimaging methods have been used to address brain metabolism. Of significant note, after entering the cells, the glucose analogue [^18^F]-FDG is phosphorylized and remains trapped in tissue in proportion to its phosphorylation rate. In the [^18^F]-FDG PET literature, both terms “brain glucose uptake” (BGU) and “cerebral metabolic rate of glucose” (CMR_glu_) have been used interchangeably. In this review, we have chosen to use the term BGU for consistency with our previously reported [^18^F]-FDG PET findings [13,14,21,22].

## 2. Transportation of Glucose and Insulin into the Brain

**Figure 1:**
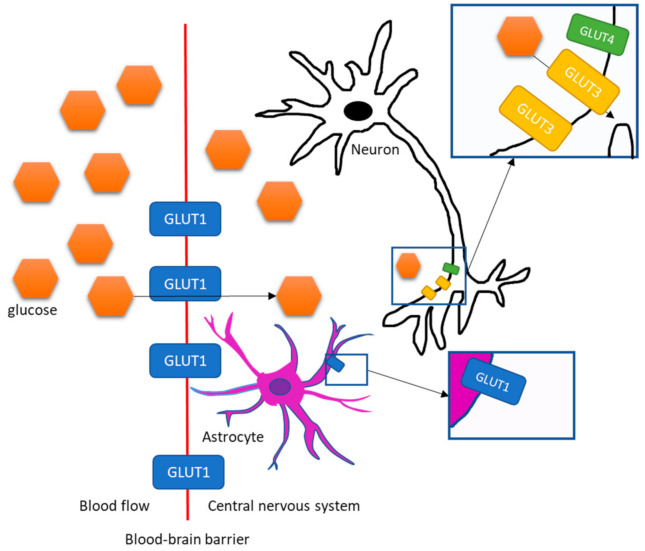
Schematic drawing of glucose (and fluorine-labelled fluorodeoxyglucose ([^18^F]-FDG)) transportation into the central nervous system and into neurons and astrocytes. Glucose is transported from the blood flow into the central nervous system by GLUT1 according to the concentration gradient of glucose, independently of insulin. GLUT1 also transports glucose into astrocytes. The main glucose transporter of neurons is insulin independent GLUT3, but some neurons also express the insulin-dependent transporter GLUT4.

Glucose uptake by cells in the human body is regulated by a family of glucose transporters, GLUTs [23,24]. The brain is a largely glucose-dependent organ, as it consumes approximately 20% of the body’s glucose supply, even though it consists only of 2% of the mass of the human body [25]. Glucose uptake from the blood flow into the central nervous system (CNS) across the blood-brain barrier (BBB) is regulated by GLUT1 [23,24] which is considered to be an insulin independent glucose transporter [26]. The same transporter also regulates glucose uptake by astrocytes, one of the glial cell types in the brain that is involved in neuroinflammation [23,26]. In contrast, neurons express mainly GLUT3 which transports glucose into neurons independently of insulin [23] (Figure 1). GLUT4s, the insulin-dependent glucose transporters, which are mainly expressed in the skeletal muscle and the adipose tissue [27], have been found also in a subset of neurons scattered across the CNS [28,29] (Figure 1). Interestingly, these neurons can be found in brain regions that are crucial for maintaining body homeostasis, such as the hypothalamus [29,30]. The importance of these GLUT4 neurons can be further highlighted by studies on mice models with insulin receptor inactivation/GLUT4 gene knock-out in specific target tissues. Kotani et al. [31] developed a mouse model with knock-out of GLUT4 in both muscle and adipose tissue by breeding mice with GLUT4 knock-out in muscles with mice with GLUT4 knock-out in adipose tissue. The tissue-specific GLUT4 knock-out models had been previously created by using Cre-LoxP gene targeting. Although these female mice were insulin resistant when compared to wild type controls, they did not develop overt hyperglycemia or diabetes by the age of 6 months [31]. Similarly, transgenic mice with inactivated insulin receptors in the muscles and adipose tissue developed IR, but not diabetes at the age of three months (the sex of the mice was not reported) [32]. In contrast, Lin et al. [29] developed a transgenic mouse model where insulin receptors were inactivated in the muscles and adipose tissue, but also in the brain, and showed that these mice were characterized by hyperglycemia, hyperinsulinemia, and IR as demonstrated during a hyperinsulinemic euglycemic clamp. By 6 months of age, 46% of male mice had developed a condition defined as diabetes (glycemia > mean + 2SD), whereas only 10% of female mice developed a similar condition by the same age. More recently, Reno et al. [33] studied brain specific GLUT4 knockout mice (8 to 16 weeks old male mice). These mice had similar GLUT4 expression in heart, muscle, and adipose tissue as wild type controls, but over 99% reduction of GLUT4 expression in the brain. In addition, these brain specific GLUT4 knockout mice developed impaired glucose tolerance and IR [33]. Thus, it seems that—although brain glucose uptake is mainly insulin independent—insulin regulates glucose uptake in the brain by a subset of neurons that may have a role in regulating whole body homeostasis.

Insulin, which is secreted from the pancreatic β-cells into the blood flow, is actively transported across the BBB in a saturable manner [34]. The traditional view has been that CNS insulin is derived through the BBB, and that insulin is not synthesized in the CNS [34,35]. However, this view has been recently challenged [36], as some rodent [37,38] and human [39] studies have discovered insulin mRNA in the brain. In addition to being actively transported across the BBB, insulin also exhibits specific functions at the BBB via the insulin receptor, such as increasing the transport of leptin, tyrosine, and tryptophane from blood to the CNS [34]. To date, it is not known whether the protein that transports insulin across the BBB and the receptor that mediates the function of insulin on BBB endothelial cells are in fact the same protein [35]. Animal studies have shown that the transportation of insulin from blood flow to the CNS can be influenced by many factors related to obesity and T2D. Obesity [40,41] and hyperglycemia [42] are associated with reduced brain insulin transport, whereas higher triglycerides (studied in starved obese mice) [43] and diabetes (when induced acutely in mice with streptozotocin) [42] seem to be associated with higher brain insulin transport. Animal studies on rats have shown that insulin receptors are widely expressed in the CNS, in particular in the olfactory bulb, the cortex, the hippocampus, hypothalamus, striatum, and cerebellum [34,44]. A small, randomized cross-over trial in humans aged 55 to 81 years showed that cerebrospinal fluid insulin levels are increased after the hyperinsulinemic clamp when compared to a placebo-saline infusion [45], and recent human research on brain glucose metabolism during the hyperinsulinemic clamp has suggested that even BGU might be partly insulin-dependent [22]. Accordingly, there is accumulating evidence of insulin having a number of specific effects in the CNS.

## 3. Central Insulin Actions

The role of insulin in the central nervous system has awoken increasing interest in the last decade, following the notion that insulin has several specific actions in the brain. Regarding the metabolic control of insulin through direct central action, there is now accumulated evidence from preclinical and recent clinical studies of a so-called “brain-liver” and a “brain-pancreas” axis. Elegant preclinical studies have shown that direct insulin action in the brain may affect endogenous glucose production (EGP) and insulin secretion [46]. More specifically, it has been shown that intracerebroventricular (ICV) injection of insulin in rats suppresses EGP, and later studies have identified the insulin-signaling pathways involved in this process [46]. Recent clinical studies seem to confirm this “brain-liver” axis as in fMRI studies, Heni et al. have shown that intranasal insulin (INI) administration on top of a low dose insulin clamp suppresses EGP in lean, but not in overweight subjects [47]. In line with these findings, our group has shown with [^18^F]-FDG-PET imaging that BGU correlates positively with EGP in morbidly obese but not in lean individuals [14]. On the contrary, INI administration during fasting did not affect EGP [48], and similarly no correlation was found between BGU and EGP in the fasting state [14]. Taken together, these data suggest that in conditions of high systemic insulin levels (like those typically seen in the post-prandial state), the brain may directly control (suppress) EGP, but this control is lost with increased adiposity.

Similarly, even though preclinical studies on dogs already showed, decades ago, that an ICV injection of insulin enhances insulin secretion [49], only recently our group showed that brain substrate handling (glucose or free-fatty acids) correlates with parameters of insulin secretion, evaluated during an oral glucose tolerance test [50]. More specifically, we have shown that BGU during the hyperinsulinemic clamp correlates with insulin secretion in non-diabetic individuals, whereas no correlation was found in 15 subjects with type 2 diabetes [50]. Moreover, brain fatty acid uptake correlated positively with insulin secretion but negatively with potentiation of insulin secretion (i.e., the augmentation of insulin secretion in the presence of potentiating factors such as the antecedent glycemia, incretins, and neural factors [50,51]). Thus, these data provide correlative evidence that in humans, the brain may be involved in the control of insulin secretion. Moreover, in two different datasets of morbidly obese subjects undergoing bariatric surgery, we showed that an increased BGU (during insulin clamp) or an enhanced brain fatty acid uptake at baseline predicts worse glycemic control at two years of follow-up [14,52], suggesting that high brain substrate uptake can predict, and perhaps lead to, metabolic deterioration in the future. Last but not least, INI has also been shown to enhance postprandial thermogenesis [53].

In addition to these effects of central insulin on whole-body homeostasis, CNS insulin also seems to play an important role in cognitive functions, and even in the neuropathological process of AD [54,55,56,57]. For example, a study on rat hippocampal slices indicated that insulin affects the formation of synapses and enhances long-term potentiation, an important part of the formation memories [58]. Moreover, animal studies have suggested that insulin might regulate the expression of neurotransmitters, such as noradrenalin [59] and acetylcholine [60]. In rats, chronic treatment with insulin administered into the third ventricle suggested that insulin regulates noradrenergic pathways in the brain [59]. In mice, the intra-peritoneal effects of insulin on cognition were suggested to be modulated by brain cholinergic pathways [60]. Of note, the current pharmacological treatment for mild to moderate AD is based on acetylcholinesterase inhibitors which inhibit the degradation of acetylcholine, thus enhancing cholinergic stimuli in the brain [61]. In addition, insulin can influence the degradation of beta-amyloid [62] and the phosphorylation of tau [63], the main constituents of neuritic plaques and neurofibrillary tangles, the neuropathological hallmarks of AD. Following the notion that central insulin seems to have a positive effect on cognition, small clinical trials on INI for the treatment of mild cognitive impairment (MCI) and mild AD have been performed [64,65,66]. These studies indicated that INI might improve cognitive functioning, but the results vary according to sex, Apolipoprotein E (APOE) genotype, and the dose of insulin administered [64,65,66,67]. In the largest study thus far—where 289 MCI or AD patients were randomized for treatment of intranasal insulin or placebo—no cognitive or functional benefits were observed in the treatment group [20].

## 4. Brain Glucose Metabolism in Alzheimer’s Disease, Mild Cognitive Impairment (MCI), Insulin Resistance and Obesity in the Fasting State Measured with [^18^F]-FDG-PET

Numerous studies have evaluated BGU in AD and MCI, but there are only few studies that have focused on the associations between IR, obesity, and [^18^F]-FDG-PET, and the results have been controversial (Table 1). The first report describing [^18^F]-FDG-PET for measuring local cerebral glucose utilization in humans was published in 1979 [68]. Since then, distinct patterns of regional cerebral hypometabolism have been shown to occur in different neurodegenerative diseases, and [^18^F]-FDG-PET is recommended for clinical use for aiding the diagnosis of memory disorders, especially in atypical cases and in the early stages of cognitive decline [69,70]. Guidelines for performing and analyzing brain [^18^F]-FDG-PET scans in the clinical setting have been published [71]. Fasting for a minimum of four hours is required before the scan. Blood glucose levels are determined before the start of the scan since hyperglycemia (≥9 mmol/L) results in reduced whole brain [^18^F]-FDG uptake due to increased competition between plasma glucose and [^18^F]-FDG [71]. Scans can be performed either as dynamic or static emission scans and started either at injection or up to 60 min post-injection [71]. Typically, after image preprocessing and reconstruction, the scans are read visually by a radiologist to detect abnormalities in regional [^18^F]-FDG uptake, but the use of semi-automated processing is recommended to assist visual reading in clinical settings [70]. For research purposes, quantitative and semi-quantitative approaches are used and, naturally, the methods used for scanning and for image analysis affect the results and limit the direct comparison of scans performed in different studies.

### 4.1. Alzheimer’s Disease

In AD, [^18^F]-FDG-PET typically shows a pattern of regional hypometabolism in the posterior cingulate cortex at early stages of the disease, and bilaterally in the temporoparietal regions at more advanced stages [72,73]. Metabolism in the occipital cortex is most often well-preserved. These regional patterns allow the differential diagnosis between AD and frontotemporal degenerations, and between AD and Lewy body disease [69,70]. [^18^F]-FDG-PET is proposed to be used as a marker of neurodegeneration in the A/T/N framework of Alzheimer’s disease which categorizes AD biomarkers according to the aspects of AD neuropathology that they reflect (A: amyloid accumulation; T: tau accumulation, or N: neurodegeneration) [74]. As glucose is the primary substrate of the synaptic function, regional cerebral hypometabolism is thought to reflect the loss of neurons in the region where hypometabolism is seen. However, a recent study suggests that part of the brain [^18^F]-FDG-PET signal can be attributed to glucose uptake by astrocytes [75]. This finding was in keeping with the astrocyte-neuron lactate shuttle, according to which glucose taken up by astrocytes is transformed into lactate, which is then transported to the neurons as energy supply [76]. In addition, downregulation of GLUT1 and GLUT3 has been shown in a neuropathological study on brains of patients with AD when compared to controls [77] which could also partly explain the brain hypometabolism detected in AD.

### 4.2. Mild Cognitive Impairment

In contrast to the hypometabolism pattern characteristic of AD, results on subjects with mild cognitive impairment (MCI) have been less consistent [78,79,80,81]. MCI can be considered a prodromal stage of AD [82,83], but significant heterogeneity exists in this diagnostic entity, and not all individuals with MCI will progress to develop Alzheimer´s disease or another form of dementia later in life [83]. A number of studies have shown that patients with MCI have hypometabolism when compared to controls in the posterior cingulate [79,84,85], and one study also suggested hypometabolism in the hippocampus [80]. In contrast, two previous studies utilizing either arterial [78] or arterialized venous blood sampling [79] and kinetic modeling of the [^18^F]-FDG images suggested that at least some MCI subjects exhibit hypermetabolism in certain brain regions such as the parietal cortex when compared to normal controls. In addition, one of these studies [78] demonstrated that MCI subjects with cortical hypermetabolism did not develop AD over a follow-up of 18 months, whereas MCI subjects with cortical hypometabolism converted to AD. Thus, the current understanding supports the notion that some MCI subjects prone to develop AD can exhibit, at least temporarily, brain hypermetabolism in order to preserve cognitive function. The cause of the transient hypermetabolism seen in MCI patients is still unknown. Furthermore, inflammatory cells in the brain utilize glucose, and neuroinflammation has been demonstrated both in MCI and AD subjects when compared to controls with PET imaging by utilizing the radiotracers [^11^C]-(R)PK11195 [86] and [^11^C]-PBR28 [87,88] that bind to 18 kDa translocator protein (TSPO), a molecule that is overexpressed by activated microglial cells in the brain [86,87,88]. One of these studies suggested an early and a late peak in neuroinflammation in the pathological process of AD [88]. It is thought that the initial inflammatory response would be protective. However, as the disease progresses, neuroinflammation might accelerate the neuropathological changes and neurodegeneration typical for AD [88]. These studies on neuroinflammation in MCI and AD, and a recent study that suggested that part of the brain [^18^F]-FDG signal could be attributed to astrocytes [75], indicate that neuroinflammation (increased microglia activation and astrocytosis) in response to accumulating beta-amyloid in the cerebral cortex could be the driver of increased brain glucose metabolism in MCI subjects at risk for developing AD dementia.

### 4.3. Systemic Insulin Resistance

A few studies have assessed the relationship between peripheral IR and fasting [^18^F]-FDG-PET. In cognitively normal elderly individuals with newly diagnosed diabetes or prediabetes, IR, measured with the homeostasis model of IR(HOMA-IR) [89] was associated with reduced cerebral metabolic rate of glucose in regions where hypometabolism is seen also in AD, i.e., in frontal, temporoparietal, and cingulate regions. The study did not report if there was a difference between the prediabetic group (*n* = 23) and the control group (*n* = 6) in glucose metabolism in these regions, possibly due to lack of power for this type of analysis [18]. Similar findings were reported also by Willette and colleagues, in cognitively normal late middle-aged subjects with a family history of AD [90]. In another large study the presence of T2D and elevated HbA_1c_ were reported to associate with brain hypometabolism [91]. In contrast, Willette et al. detected an association between higher HOMA-IR and hypermetabolism in the medial temporal lobe of individuals with MCI who later progressed to AD. In patients already diagnosed with AD, a higher HOMA-IR was associated with lower [^18^F]-FDG uptake [16] (Table 1).

### 4.4. Obesity

Whereas we have previously described no difference in fasting BGU between obese and lean individuals using graphical analysis (a quantification method based on a dynamic PET scan and frequent arterialized blood sampling) [22], a recent large study reported a positive correlation between BMI and fasting BGU, in 168 cognitively unimpaired elderly from the Alzheimer’s disease neuroimaging initiative (ADNI) database. BGU in this study was evaluated as standardized uptake value (SUV) ratio, using the SUV from pons-vermis area as the reference region. This region is typically used in AD research because it is not affected by hypometabolism [92]. Another study also using the ADNI database and reporting statistical parametric mapping T-values also reported brain hypermetabolism in obese women, but not in men [93] (Table 1). Our findings on no association between obesity and brain glucose metabolism are supported by a study on mice [94] which were fed a high-fat diet. When compared to mice with normal diet, the high-fat diet fed mice showed reduced GLUT1 expression and reduced [^18^F]-FDG uptake at the BBB after only 3 days. However, both BBB GLUT1 expression and [^18^F]-FDG uptake were normalized in a prolonged situation, i.e., after 21 days of high-fat diet [94].

Taken together, it emerges that there is inconsistency in the published literature regarding the effects of obesity and IR on brain glucose metabolism. Considering that these conditions are closely linked, it would be expected that both obesity and IR would show similar associations with brain glucose metabolism. However, the few studies published thus far suggest that IR would be associated with brain hypometabolism (except in the MCI stage) whereas obesity would be associated with hypermetabolism in cognitively normal elderly (Table 1). Of note, the methods to analyze [^18^F]-FDG vary across studies. In studies focusing on neurodegeneration and cognitive decline, [^18^F]-FDG uptake is commonly reported as a ratio that has been normalized to a reference region (such as the pons or the global mean standard uptake value of the individual scan) to decrease noise and to highlight regional changes in brain glucose metabolism when compared to the basal [^18^F]-FDG signal. In contrast, studies by metabolically focused groups typically report the actual quantified brain glucose uptake rate which requires frequent blood sampling during the scan [95]. These differences in reporting results from [^18^F]-FDG PET studies may be confusing especially to readers who are not familiar with the PET methodology and could perhaps partly explain the discrepancy considering current knowledge on the associations between obesity, IR, and BGU. Another possibility is that the contrasting results would depend on the differences in the study populations regarding, for example, the age and cognitive status of the participants.

**Table 1:** Summary of fluorine-labelled fluorodeoxyglucose ([^18^F]-FDG) studies evaluating the effect of Alzheimer’s disease (AD), mild cognitive impairment (MCI), aging, obesity, and insulin resistance (IR) on brain glucose metabolism during fasting and insulin clamp ^#^.

	N	Age (Years)	Subjects’ Status	BGU	Method Used	Reference
*fasting*						
AD ^§^	548	50–85	199 AD, 114 MCI, 98 FTD, 27 DLB, 110 controls	decreased	3-D Z scores	Mosconi et al. [71]
MCI	96	69 ± 4.6	8 MCI, 66 AD, 22 controls	decreased	count ratio	Minoshima et al. [80]
MCI	47	73 ± 8.1	17 MCI, 17 AD, 13 controls	decreased	SUV ratio	Bailly et al. [83]
MCI	27	66 ± 10	10 MCI, 9 AD, 8 controls	increased/decreased	quantification *	Ashraf et al. [77]
MCI	63	76.9 ± 5.8	20 MCI, 19 AD, 24 controls	increased/decreased	quantification *	Croteau et al. [78]
Aging	205	20–82	cognitively normal adults	decreased	count ratio	Goyal et al. [96]
Obesity	29 168 222	45 ± 9 74 ± 6 74 ± 6	cognitively unimpaired cognitively unimpaired cognitively unimpaired	unchanged increased increased	quantification * SUV ratio SPM T-ratio	Tuulari et al. [22] Pegueroles et al. [91] Sala et al. [92]
IR	29 150	74 ± 7 61 ± 6	cognitively normal cognitively normal with family history of AD	decreased decreased	counts count ratio	Baker et al. [18] Willette et al. [89]
*euglycemic insulin clamp*						
AD	-	-	-	-	-	-
MCI	-	-	-	-	-	-
Aging	194	20–80	cognitively unimpaired	decreased	quantification *	Rebelos et al. [21]
Obesity	34	45 ± 9	obese and lean	increased	quantification *	Tuulari et al. [22]
IR	194	20–80	cognitively unimpaired	increased	quantification *	Rebelos et al. [21]

^#^ AD: Alzheimer’s disease; MCI: mild cognitive impairment; FTD: frontotemporal dementia; DLB: dementia with Lewy bodies; IR: insulin resistance. Age entries are mean ± SD, or range as reported in the original publications. ^§^ There is a consensus according to multiple studies on different study populations that there is hypometabolism in AD. * The exact methods of quantification used were the following: Ashraf et al.: spectral analysis using arterial input function; Rebelos et al.: fractional uptake rate using “arterialized” input function; Tuulari et al. and Croteau et al.: graphical analysis (Patlak plot) using “arterialized” input function.

## 5. Insulin-Stimulated BGU

From a metabolic standpoint, studies addressing the possible effect of insulin on brain glucose metabolism have either used the insulin clamp; used somatostatin in conjunction with glucose and insulin infusion to assess the baseline insulin effect; or have directly given insulin centrally through intranasal insulin administration. Studies evaluating BGU under more physiologic stimuli (for instance during feeding) are scanty.

**Figure 2:**
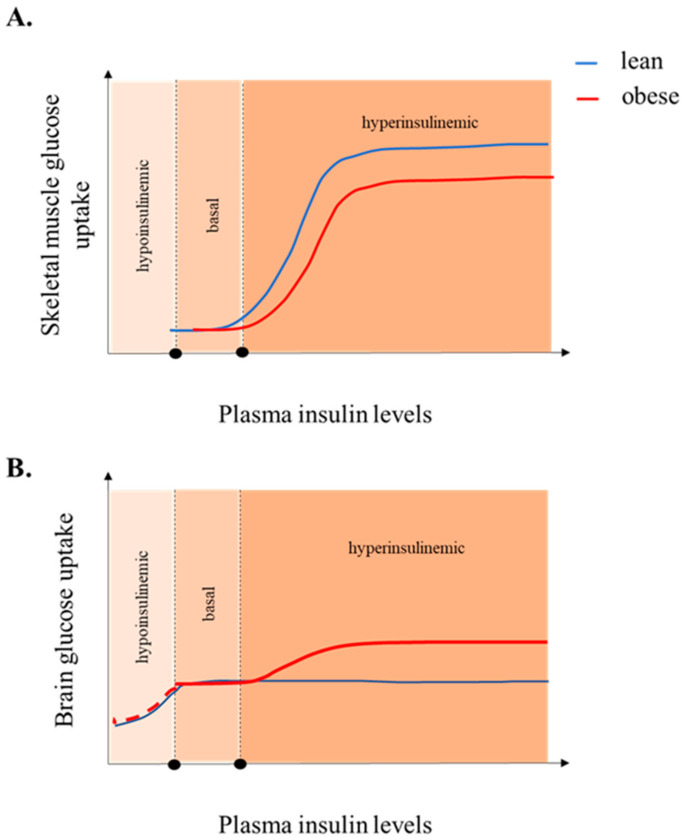
The available literature suggests that in the brain, the dose-response curve of insulin is differentially affected based on adiposity. Thus, in lean subjects the curve is shifted to the left (**B**) when compared to skeletal muscle uptake (**A**) (as basal insulin levels increase BGU, but further increasing of the insulin concentrations above the fasting insulin levels does not yield an increase in BGU), whereas in obese subjects systemic hyperinsulinemia (insulin clamp) results in increase in BGU (**B**). In the obese, the dashed line in the hypoinsulinemic area is to point out that the effect of basal insulin levels on BGU has not been investigated in obese individuals and thus the dashed line is hypothetical (**B**). Skeletal muscle glucose uptake in obese and lean is given as a way of comparison based on Bonadonna et al. [102] (**A**).

Early studies on healthy young controls indicated that insulin stimulation had no effect on BGU, measured with [^18^F]-FDG-PET during a hyperinsulinemic euglycemic clamp [97]. In contrast, a later study on metabolically healthy males that first suppressed endogenous insulin production below basal levels with a somatostatin infusion and then compared BGU with and without infused external insulin concluded that during insulin infusion, BGU was significantly higher when compared to the state with very low circulating insulin [98]. These contrasting results could, according to the authors of the latter study [98], be explained by the possibility that insulin-stimulated BGU has already reached a maximum at physiological basal insulin levels. However, when insulin secretion is suppressed to concentrations below normal, an effect of infused insulin on BGU is seen (Figure 2B). The authors concluded that apparently, the dose-response curve of brain insulin-stimulated uptake is shifted to the left of the dose-response curves of other insulin-sensitive organs in the body (such as muscles and the liver) (Figure 2A), meaning that most probably, BGU is already maximally stimulated at basal insulin conditions in healthy adults, which is why no effect of an additional insulin stimulation can be seen [97]. In addition to the two studies on healthy subjects mentioned above, several studies from Turku PET Centre evaluated BGU during a hyperinsulinemic clamp in different metabolic conditions. These studies have shown that in conditions of systemic IR due to multiple etiologies (obesity, impaired glucose tolerance status, carriers of the AKT2-mutation who are genetically predisposed to IR), insulin-stimulated BGU, measured during the hyperinsulinemic euglycemic clamp, is increased when compared to lean, metabolically healthy controls [12,22,99]. Moreover, in the study by Tuulari and colleagues, it was clearly shown that BGU was enhanced during the insulin clamp as compared to the fasting BGU in the morbidly obese, but not in the lean participants [22]. This finding has been later shown also in animals [100]. Interestingly, the increased insulin-stimulated BGU seen in obese, insulin resistant individuals is reversible, since following weight loss, the difference between fasting and insulin-stimulated BGU was no longer found [22]. Recently, in the largest study thus far evaluating the predictors of BGU during conditions of insulin clamp, it emerged that insulin sensitivity (indexed by the M value of the insulin clamp), is the best predictor of insulin-stimulated BGU compared to several other parameters evaluated such as age, gender, BMI, plasma insulin levels, and inflammatory markers [21]. Presence of T2D further contributes to higher BGU. Considering that in recent years the statistical power of neuroimaging studies has been questioned [101], this study essentially confirmed the previous reports.

## 6. Attempts to Define Central Insulin Resistance

The definition of systemic insulin resistance is based on tissue-level studies where several molecular defects have been established [5,102]. For obvious reasons, such studies cannot be performed in the human brain in vivo to define central IR. Considering that both systematic and central nervous system IR have been linked to cognitive decline and AD, and to the dysregulation of body homeostasis, many groups have attempted to demonstrate IR in the human brain with different methods. A seminal post-mortem study by Talbot and colleagues demonstrated IR at tissue level in the brains of patients with AD. They showed that in the neurons of AD brains the response to insulin incubation by the neuronal insulin receptors and the signaling cascades following insulin receptor activation was attenuated when compared to cognitively normal controls and MCI subjects, and that these differences were independent of diagnosis of T2D before death [6]. More recently, a group led by Kapogiannis used neuronally derived extracellular vesicles extracted from plasma to demonstrate defects in insulin signaling in AD patients when compared to controls [103,104] and also in patients with bipolar depression [105]. Most studies trying to address whether there is central IR in the context of systemic IR have used neuroimaging methods, such as PET, functional magnetic resonance imaging (fMRI) and magnetoencephalography (MEG). Therefore, the definition of brain IR has varied depending on the implemented method: i.e., a blunted cerebrocortical insulin effect in obese individuals when compared to lean when MEG is applied [106]; decreased intranasal insulin-induced suppression of hypothalamic blood flow in fMRI [15]; or an insulin-induced increase in brain glucose uptake (BGU) in [^18^F]-FDG-PET studies [13,99]. However, since multiple measures with different neuroimaging modalities are typically not used within a single study, the integration of these results is difficult. We recently reported that similar findings can be found with PET and fMRI studies when addressing associations between central insulin action and visceral fat mass. Thus, although findings from one neuroimaging method might be translated into the findings of another, more studies combining different approaches on the same study subjects are needed to establish the relationship between different features of brain IR and their functional consequences [13]. Of note, brain magnetic resonance spectroscopy (MRS) using a proton (^1^H-MRS) or a carbon nucleus (^13^C-MRS) is yet another useful neuroimaging technique which can evaluate several metabolites including glucose [107] and glycogen [108] levels, and thus can be of paramount importance when evaluating brain metabolism.

## 7. Increased Brain Glucose Uptake and Brain Insulin Resistance, Two Sides of the Same Coin?

Taken together, the literature reviewed above, multiple studies in both animals and humans have demonstrated that during an insulin clamp, the human brain operates in an opposite way than skeletal muscle and/or adipose tissue. In these tissues it is well established that insulin-stimulated glucose uptake is markedly reduced in insulin resistant subjects, when compared to insulin sensitive controls. From an alternative point of view, it could also be argued that in fact, in contrast to the lean subjects, in obese subjects the brain responds to the hyperinsulinemic stimulation similarly as the muscles and the adipose tissue, i.e., that externally administered insulin induces glucose uptake by the brain. The molecular mechanisms that lead to this peculiar characteristic of brain metabolism in obesity are thus far not understood, but several hypotheses have been put forward. Bahri and colleagues have suggested that IR does not have an effect on the expression of GLUT transporters in the brain, whereas their expression is decreased in skeletal muscle in IR [99]. In keeping with this, Kobayashi et al. have shown that although fasting and diabetes markedly decreased GLUT4 expression in adipose tissue, brain GLUT4 expression was only marginally affected by the same conditions [109]. However, in this case it would be assumed that BGU would be greater during the hyperinsulinemic clamp, also in lean, and not only in obese individuals. Our group is currently investigating whether the increased BGU in IR is driven by central inflammation (NCT04343469), based on the recent finding that brain [^18^F]-FDG uptake is partly driven by astrocytes [75], and by the animal studies showing that in rats, a high-fat diet leads to astrocyte proliferation and activation (called astrogliosis) [110]. Other possible explanations could be that since both obesity and T2D are linked to damage at the BBB, the entry of glucose into the CNS could be facilitated by the disruption of the BBB. Nevertheless, this hypothesis does not explain the differences demonstrated between the fasting and insulin stimulated states in BGU in the obese. Moreover, it is well-established that insulin has vasodilating effects, and that for instance in skeletal muscle insulin-mediated vasodilation is coupled with enhanced glucose uptake [111]. However, to the best of our knowledge, this coupling has not been demonstrated in the human brain. Actually, in a small human PET study, we showed that insulin clamp does not affect brain blood flow in normal glucose tolerant or impaired glucose tolerant individuals [99].

It has been demonstrated that in obese individuals the dose-response curve of whole-body glucose uptake according to increasing plasma insulin is both attenuated and shifted to the right when compared to lean individuals [102] (Figure 2A). In addition, the study by Bingham et al. [98] that demonstrated insulin having an effect on BGU in metabolically healthy subjects only when basal insulin levels were lowered to below physiological levels, suggested a shift to the left of the brain glucose uptake curve (when compared to glucose uptake in the skeletal muscles and the adipose tissue). Considering these findings, it could be possible that in the obese, insulin-resistant individuals, BGU is not saturated at basal insulin levels, possibly because of a shift towards the right in the insulin-stimulated brain glucose uptake dose-response curve when compared to lean individuals, in a similar manner as happens with the whole-body glucose response curve (Figure 2B). Accordingly, we speculate that BGU during the hyperinsulinemic clamp could reflect GLUT4-mediated, i.e., insulin-dependent BGU by the subset of neurons that contain this insulin-dependent glucose transporter. This theory is supported by the study on mice which lacked insulin receptors in the GLUT4 neurons in the brain [29]. In contrast to the findings in humans and minipigs showing hypermetabolism during the hyperinsulinemic clamp in the obese, there was no increase in BGU during the hyperinsulinemic clamp in these obese mice, when measured with [^3^H]-FDG autoradiography directly after the clamp [29]. We realize however, that even though this theoretical hypothesis provides an explanation for the increase in BGU in obese individuals during clamp conditions when compared to the fasting state, it does not explain why obese individuals have higher BGU than the lean ones during clamp conditions. Possibly, this difference could be due to for example a difference between lean and obese in the main glucose transporter in the CNS, GLUT1, or to a difference in the amount of insulin that enters the brain during the hyperinsulinemic clamp. To conclude, this theory will need to be verified by future studies that would for example compare the expression of the different GLUTs in the CNS between obese and lean subjects. To date, a difference between lean and obese subjects in BBB GLUT1 expression has only been demonstrated in high-fat diet fed mice [94], as explained in paragraph 4.4.

## 8. Conclusions and Future Directions

There is mounting evidence that insulin affects brain glucose metabolism, and several changes in central insulin action have been documented in the context of systemic IR, suggestive of central IR. However, the role of insulin on brain glucose metabolism remains to be fully established. In our view, two steps of “harmonization” of the current knowledge are warranted: (a) closing the gaps in the interpretation of the different results yielded from the different neuroimaging studies (MEG, fMRI, PET) which have tried to define central IR with neuroimaging and (b) joint efforts between metabolic and neurologic researchers to advance the understanding of the links between metabolic and neurodegenerative disorders.

The human brain is one of the most understudied organs, for obvious difficulties in acquiring tissue samples. Neuroimaging studies constitute the basis of characterization of metabolic, inflammatory, and neurodegenerative processes of the human brain in vivo. However, they are expensive, and only a few specialized centers across the world combine both clinical and research neuroimaging. To battle these problems, and to assist the advancement of understanding the alterations between metabolic and neurologic disorders, common, shared databases of PET imaging and of metadata of basic clinical characteristics (including age, gender, BMI, plasma glucose, and insulin levels) would be warranted. Similar large depositories of functional MRI data, and of PET images to study AD and MCI, including fasting [^18^F]-FDG-PET images already exist (Neurosynth and ADNI databases). Databases on more diverse populations, including young and middle-aged subjects, could broaden the understanding of a life-course perspective on how obesity and systemic and CNS IR are linked to neurodegeneration. Moreover, neurologic and metabolic researchers should join efforts to thoroughly characterize their subjects to both ends. For instance, metabolic studies have typically not been assessing cognitive function in their studied subjects, and to the best of our knowledge more advanced metabolic studies (like the insulin clamp in conjunction with [^18^F]-FDG-PET imaging) have never been applied in complying subjects with MCI or AD. Longitudinal studies with long follow-up will define whether the increased insulin-stimulated BGU in the context of IR precedes cognitive decline, and/or further aggravation of the metabolic status. We believe that bridging metabolic and neurologic research is an important opportunity to follow, and the expected gained knowledge can help tackle the epidemic of neurodegenerative disorders and increase our understanding of the pathophysiology of IR.
